# Supplementary material for: Content-rich biological network constructed by mining PubMed abstracts
Source: BMC Bioinformatics. 2004 Oct 8;5:147. doi: 10.1186/1471-2105-5-147 (PMC528731; doi:10.1186/1471-2105-5-147)
Supplement: Additional File 2 — The original results of the above study (non-essential files are deleted to keep the file size under the limit set by BMC bioinformatics). [file 1471-2105-5-147-S2.bz2 › chilibotAdditionalFile2/dip05/718ID9159523E18055/html/RSEA_RSEB.html]

 


 **RSEA** and **RSEB** 
  
Found 4 abstracts in PubMed, retrieved 4.  
 

 What does Google say? 
 PDF only 
| .edu only 

---

**Interactive relationship** (e.g. stimulation, inhibition, etc)

**Neutral relationship**- **RseB**  binding to the periplasmic domain of  **RseA**  modulates the  **RseA**  sigmaE interaction in the cytoplasm and the availability of sigmaE.RNA polymerase.  Ref: 11777003 J Biol Chem, 2000
- The C terminal domain of  **RseA**  interacts with  **RseB** , a periplasmic protein.  Ref: 11777003 J Biol Chem, 2000
- **RseB**  is a periplasmic protein and was found to co purify with the periplasmic domain of  **RseA** , indicating that  **RseB**  probably exerts negative activity on sigma E through  **RseA** .  Ref: 9159523 Mol Microbiol, 1997
- **RseB**  is a periplasmic protein, which negatively regulates sigma E activity and specifically interacts with the C terminal periplasmic domain of  **RseA** .  Ref: 9159522 Mol Microbiol, 1997
- **RseB**  binding to the C terminal domain of  **RseA**  increases the affinity of  **RseA**  for sigmaE by 2 to 3 fold Kd 50 100 nM.  Ref: 11777003 J Biol Chem, 2000

**Non-interactive relationship** (e.g. studied together, co-existance, homology, etc.)

- Modulation of the Escherichia coli sigmaE RpoE heat shock transcription factor activity by the  **RseA** ,  **RseB**  and RseC proteins.  Ref: 9159522 Mol Microbiol, 1997
- The sigmaE mediated response to extracytoplasmic stress in Escherichia coli is transduced by  **RseA**  and  **RseB** , two negative regulators of sigmaE.  Ref: 9159523 Mol Microbiol, 1997
- Purification from crude cellular extracts yields cytoplasmic, soluble sigmaE RNA polymerase as well as membrane sequestered sigmaE. **RseA**  and sigmaE. **RseA** . **RseB** .  Ref: 11777003 J Biol Chem, 2000
- The relative concentration of sigmaE  **RseA**   **RseB**  is 2 5 1 and this ratio remains unaltered upon heat shock induction of the sigmaE regulon.  Ref: 11777003 J Biol Chem, 2000
- Such multiple effects were observed in mutants with trans acting  **rseA** ,  **rseB** , rseC, RseD and cis acting H1 1 and H1 2 mutations, but not in mutants in which SerH is affected developmentally B2092, B2101, B2103, B2107.  Ref: 1732170 Genetics, 1992
- Under induction conditions, strains lacking  **RseB**  and or C show wild type induction of sigma E activity, indicating either the presence of multiple pathways regulating sigma E activity, or the ability of  **RseA**  alone to both sense and transmit information to sigma E.  Ref: 9159523 Mol Microbiol, 1997
